# Burnout among midwives and attitudes toward midwifery: A cross-sectional study from Baden-Württemberg, Germany

**DOI:** 10.18332/ejm/150582

**Published:** 2022-07-29

**Authors:** Nicolas Paul, Marcus Limprecht-Heusner, Jutta Eichenauer, Christel Scheichenbauer, Till Bärnighausen, Stefan Kohler

**Affiliations:** 1 Heidelberg Institute of Global Health (HIGH), Faculty of Medicine and University Hospital, Heidelberg University, Heidelberg, Germany; 2 Hebammenverband Baden-Württemberg, Backnang, Germany; 3 Institute of Social Medicine, Epidemiology and Health Economics, Charité - Universitätsmedizin Berlin, corporate member of Freie Universität Berlin and Humboldt-Universität zu Berlin, Berlin, Germany

**Keywords:** burnout, Germany, job resignation, mental health, midwives, stress

## Abstract

**INTRODUCTION:**

Midwifery services are a cornerstone of maternal care, but the mental health of midwives is at risk in many work settings. The aim of this study was to assess burnout and attitudes toward midwifery among midwives in Baden-Württemberg, Germany.

**METHODS:**

A cross-sectional online survey among midwives was conducted from 16 October to 10 December 2017. Burnout was assessed using the Copenhagen Burnout Inventory (CBI).

**RESULTS:**

A total of 602 survey respondents were studied; 48.3%, 38.2%, and 23.3% of midwives reported moderate or high (CBI score ≥50) levels of personal burnout, work-related burnout, and client-related burnout, respectively. Midwives with moderate or high burnout in at least one CBI dimension worked more weekly hours, were more commonly employed, and worked more frequently in the hospital. In turn, midwives with low burnout levels worked fewer weekly hours, more commonly freelance, and more frequently community based (all p<0.001). Moderate or high burnout levels were associated with a reduced likelihood to recommend midwifery as a profession (OR=0.34; 95% CI: 0.23–0.49) and an increased likelihood to intent leaving the profession (OR=3.39; 95% CI: 2.0–5.9) in a multivariable regression adjusting for midwife characteristics and work practices.

**CONCLUSIONS:**

Burnout symptoms were common among midwives. Burnout could be a health risk for midwives and a challenge to the profession by discouraging present and future midwives from practicing midwifery.

## INTRODUCTION

The availability of midwifery services has been identified as a cornerstone of high-quality maternal care^1^. The provision of midwifery services was shown to have a positive effect on several outcomes of maternal care, including reductions in maternal and neonatal mortality, improved psychosocial outcomes, reductions in infections, and a shorter hospital stay for newborns^2^. During their practice, midwives can feel occupational stress, with, for instance, 7.2% to 22.1% of Swedish and Australian midwives reporting moderate to very severe levels of stress^3,4^. Midwives appear also at risk for mental health problems, such as post-traumatic stress disorder due to traumatic perinatal events in up to 36% of midwives in the United States^5^, or anxiety in 8.6% to 38% of midwives in Sweden, Australia and the United Kingdom^3,4,6^, or depression in 9.6% to 33% of midwives in Turkey, Sweden, Australia and the United Kingdom^3,4,6,7^. Moreover, midwives frequently report symptoms of burnout^8-10^.

Burnout occurs as a response to permanent, job-related emotional and interpersonal stressors and can be defined as a combination of exhaustion, cynicism, and inefficacy^11^. Burnout makes midwives more likely to leave their profession^12^, more likely to take sick leave^13^, and impairs their quality of life^12^. The two instruments most commonly used to assess burnout are the Maslach Burnout Inventory, which includes 22 statements that relate to emotional exhaustion, depersonalization, and reduced personal accomplishment^14^, and the Copenhagen Burnout Inventory (CBI), which includes 19 questions that relate to personal burnout, work-related burnout, and client-related burnout^15^.

A recent systematic review^9^ on the prevalence of burnout among midwives identified 14 studies from Australia^3,16-19^, New Zealand^20^, Norway^13^, Sweden^21^, Denmark^15,22^, the United Kingdom^6^, and Canada^12^ that utilized the CBI. Across studies, the systematic review reported a pooled prevalence of 50% for personal burnout, 40% for work-related burnout, and 10% for client-related burnout. Factors that were associated with higher CBI scores were lower age, being single, lack of staff and resources, low salary, negative work environments, as well as poor professional recognition and organization. Lower levels of burnout were reported for midwives working in rural areas, for midwives with extensive experience, and for midwives with high levels of autonomy^9^. Furthermore, midwives working in midwife-centered caseload models of care, in which a known midwife cares for a woman throughout the maternity continuum^23^, had lower burnout rates than midwives working in other care models^9^, for instance, in shift-based work in a hospital’s maternity department^18,20,22^. Lower burnout rates among midwives working freelance and in caseload models have been explained by higher levels of autonomy, empowerment, professional recognition, and more meaningful relationships with clients^20,24^.

Most studies measuring burnout among midwives have been conducted in caseload and non-caseload models of care in Australia, Scandinavia, the United Kingdom, Japan, Spain, or Turkey^8-10^. However, there are substantial differences in midwifery practice, between and within countries, with respect to the role and responsibilities of midwives, midwifery education, organization, and care models^2,23^. To our knowledge, the present study and our related study^25^ are the first to report burnout rates for midwives in Germany, where caseload and other work models co-exist.

The aims of the present study were: 1) to assess the prevalence of personal, work-related, and client-related burnout among midwives based on an online survey of midwives in the German state of Baden-Württemberg; 2) to identify midwife-related and work-related correlates of burnout; and 3) to investigate the association of burnout and recommending midwifery as a profession as well as having intentions to leave the profession. Our related study compared self-reported health, well-being, total burnout levels, and job satisfaction between freelance midwives, employed midwives, and midwives working in caseload and non-caseload models^25^.

## METHODS

### Study design and data collection

A cross-sectional online survey of midwives was conducted in collaboration with the Midwifery Association Baden-Württemberg (*Hebammenverband Baden-Württemberg*) and a round table for midwifery and obstetric care (*Runder Tisch Geburtshilfe*) in Baden-Württemberg, Germany. The survey was part of a broader federal health services assessment in midwifery and obstetric care, which also comprised surveys with midwifery students, early mothers and parents, and hospitals providing midwifery and obstetric care^26,27^. In the midwives’ online survey, some questions were asked to all midwives, other questions were tailored based on a midwife’s work models and whether a midwife was actively practicing midwifery.

The survey collected data about midwives’ work experiences, services, and attitudes toward midwifery (e.g. intention to leave midwifery and recommending midwifery as a profession). Additionally, the survey asked midwives about their well-being, health, and work satisfaction. To assess burnout, the CBI was translated into German (Supplementary file Box S1) and included in the online survey. The survey was open to all midwives, but it was promoted only in the state of Baden-Württemberg. The Midwifery Association Baden-Württemberg invited midwives to participate in the study through its network and social media platforms. There were 2410 freelance midwives and 1476 hospital-employed midwives in Baden-Württemberg in 2017, according to data from the National Association of Statutory Health Insurance Funds^28^. The number of midwives in Germany who practice midwifery at a certain point in time is, however, uncertain. Participation was voluntary, anonymous, and possible from 16 October to 10 December 2017.

### Copenhagen Burnout Inventory

The CBI assesses fatigue and exhaustion and consists of three dimensions: personal burnout (6 questions), work-related burnout (7 questions), and client-related burnout (6 questions). Personal burnout is the physical and psychological fatigue and exhaustion irrespective of occupation. Work-related burnout is fatigue and exhaustion with respect to a person’s work. Client-related burnout is fatigue and exhaustion with respect a person’s work with clients^15^. Questions are answered on a 5-point Likert scale; 12 questions with frequency responses (always, often, sometimes, seldom, never/almost never) and 7 questions with intensity responses (to a very high degree, to a high degree, somewhat, to a low degree, to a very low degree). Answers are transformed to a score between 0 and 100. For each burnout dimension, an average score is calculated. Higher scores indicate higher levels of burnout. In line with other studies using the CBI, we interpret CBI scores of ≥50 and <75 as moderate burnout, and ≥75 and <100 as high burnout. CBI scores of 100 have previously been interpreted as severe burnout but did not occur in our study. While some previous studies categorized CBI scores <50 as no burnout, we interpret scores of ≥25 and <50 as low burnout and scores <25 as lowest burnout^3,6,9,12,16,29^.

### Midwifery practice and training in Germany

German regulations require any birth to be accompanied by a midwife^30^. Midwives also assist women with antenatal care, postnatal care, and during infancy. Midwives autonomously support women with low-risk pregnancies, often without the presence of a medical doctor, whereas obstetricians assist women with high-risk pregnancies. Since 1985, midwives in Germany completed a three-year, secondary level vocational training at schools of midwifery that were affiliated to hospitals. Midwifery training, however, has been shifting towards university-based Bachelor’s degree programs within a European harmonization stipulated by a 2013 European Union directive^30^. Vocational training and university-based training co-exist for a transitional period until 2027 [§77 Hebammengesetz (midwives act)].

Several models of practicing midwifery are possible in Germany^30^. Combinations of work models are common and transitions between part-time and full-time work are frequent. A key difference between work models is whether a midwife is employed, self-employed as a freelance midwife, or both. Related to their work model, midwives may work in hospitals and/or community-based settings. Those midwives working in hospitals are either employed by the hospital and work in shifts, or use hospital facilities as an external, freelance midwife (*Beleghebamme*). The cooperation of external, freelance midwives, who are affiliated with a hospital, is based on individual contracts. Midwives who work community-based and without hospital affiliation may work freelance and assist home births or work either employed or freelance in midwife-led, community-based birth centers. Freelance midwives can, in principle, provide all services to offer continuity in pregnancy, maternal, and newborn care. Only 13969 (1.8%) of 776306 births in Germany took place outside of hospitals in 2020^31^. Related to high in-hospital delivery rates, growing concentration of hospital care, and raised insurance premium for birth assistance, only 4614 (25%) of 18335 freelance midwives assisted births in 2020^32^.

### Data analysis

The analyzed study sample was generated by excluding surveys from midwives who did not practice midwifery in the year prior to the study and surveys with missing values for the study variables. For each burnout dimension, a CBI score was derived from the individual CBI questions^15^. The internal reliability of the CBI scores was assessed using Cronbach’s alpha. Missing answers to CBI questions were replaced by the respective respondent’s mean CBI scores within a burnout dimension if three or fewer CBI answers were missing overall and two or fewer answers were missing within the respective burnout dimension. Mean CBI scores were visualized in histograms and radar charts. Characteristics and work practices of midwives were compared by burnout level (low burnout in all dimensions versus moderate or high burnout in at least one dimension). Pearson’s χ^2^ tests were used to assess differences in categorical variables. Two sample t-tests were used to assess differences in continuous variables. After describing midwives’ characteristics and work practices by burnout level, we assessed the relationship of moderate or high burnout in at least one CBI dimension and other factors with attitudes toward midwifery. Specifically, we investigated midwives’ attitudes towards recommending midwifery as a profession and their intention to leave midwifery in univariable and multivariable logistic regression analyses. The statistical significance level was p<0.05. All analyses were conducted in Stata, version 15.1 SE.

## RESULTS

### Midwives and work practices

Of 722 surveys submitted, 628 were from midwives who practiced midwifery in the year prior to the study. For 33 midwives, one of 19 answers to the CBI questions was missing. For 13 midwives, between 2 and 19 answers to the CBI questions were missing. The final study sample included 602 datasets after replacing missing CBI scores and excluding datasets with remaining missing values for the study variables.

More than half of the midwives in the study sample were either aged 35–44 (26.9%) or 45–55 (32.4%) years and received 12 to 13 years of schooling (63.0%). Most midwives were part of a midwifery association (93.9%) and practiced mostly in the federal state of Baden-Württemberg (97.0%). Professional experience varied from <4 years (16.9%) to >25 years (23.9%). The majority of midwives (82.4%) did not intend to leave midwifery within 5 years. Midwives in our sample practiced midwifery in a variety of work models: 45.3% worked freelance without hospital affiliation, 7.1% worked freelance with hospital affiliation, 26.4% were employed, and 21.1% were both employed and worked freelance. A share of 45.8% worked only community based, 17.3% only in the hospital, and 36.9% in both settings. Over half (60.6%) of surveyed midwives attended births. Weekly work hours varied from <24 hours (21.3%) to >55 hours (7.0%). Midwives worked in rural areas (36.0%), small cities (21.6%), medium-sized cities (18.8%), or large cities (23.6%) (Tables 1 and 2).

**Table 1:** Characteristics of midwives participating in a survey of midwives in Baden-Württemberg, Germany, 2017 (N=602)

*Characteristics*	*All midwives n (%)*	*Midwives with low burnout n (%)*	Midwives with moderate or high burnout* n (%)	*p*
**Total**, n	602	271	331	
**Age** (years)				0.073
≤24	20 (3.3)	9 (3.3)	11 (3.3)	
25–34	132 (21.9)	45 (16.6)	87 (26.3)	
35–44	162 (26.9)	76 (28.0)	86 (26.0)	
45–54	195 (32.4)	97 (35.8)	98 (29.6)	
≥55	93 (15.4)	44 (16.2)	49 (14.8)	
**Education level** (years)				0.50
9–10	167 (27.7)	71 (26.2)	96 (29.0)	
12–13	379 (63.0)	176 (64.9)	203 (61.3)	
Midwifery studies	23 (3.8)	7 (2.6)	16 (4.8)	
Nursing or health sciences studies	7 (1.2)	4 (1.5)	3 (0.9)	
Other studies	26 (4.3)	13 (4.8)	13 (3.9)	
**Completed additional training**				0.73
No	211 (35.0)	93 (34.3)	118 (35.6)	
Yes	391 (65.0)	178 (65.7)	213 (64.4)	
**Midwifery association member**				0.008
No	37 (6.1)	9 (3.3)	28 (8.5)	
Yes, Midwifery Association Baden-Württemberg	519 (86.2)	246 (90.8)	273 (82.5)	
Yes, other midwifery association	46 (7.6)	16 (5.9)	30 (9.1)	
**Practicing mostly in Baden-Württemberg**				0.60
No	18 (3.0)	7 (2.6)	11 (3.3)	
Yes	584 (97.0)	264 (97.4)	320 (96.7)	
**Professional experience as a midwife** (years)				0.68
≤4	102 (16.9)	41 (15.1)	61 (18.4)	
5–9	89 (14.8)	37 (13.7)	52 (15.7)	
10–14	111 (18.4)	55 (20.3)	56 (16.9)	
15–19	80 (13.3)	40 (14.8)	40 (12.1)	
20–24	76 (12.6)	34 (12.5)	42 (12.7)	
≥25	144 (23.9)	64 (23.6)	80 (24.2)	
**Personal burnout score**	46.4 (17.6)	31.3 (10.1)	58.7 (12.0)	<0.001
**Work-related burnout score**	43.0 (15.6)	30.8 (9.7)	53.0 (12.1)	<0.001
**Client-related burnout score**	33.8 (18.7)	21.1 (12.2)	44.1 (16.6)	<0.001
**Average burnout score**	41.1 (15.6)	27.8 (8.6)	52.0 (10.8)	<0.001
**Recommending midwifery as a profession**				<0.001
No	51 (8.5)	8 (3.0)	43 (13.0)	
Rather no	200 (33.2)	66 (24.4)	134 (40.5)	
Rather yes	216 (35.9)	109 (40.2)	107 (32.3)	
Yes	135 (22.4)	88 (32.5)	47 (14.2)	
**Intention to leave midwifery**				0.003
No	496 (82.4)	242 (89.3)	254 (76.7)	
In 1 year or sooner	42 (7.0)	14 (5.2)	28 (8.5)	
In 2 years	9 (1.5)	1 (0.4)	8 (2.4)	
In 3 years	14 (2.3)	4 (1.5)	10 (3.0)	
In 4 years	19 (3.2)	5 (1.8)	14 (4.2)	
In 5 years	22 (3.7)	5 (1.8)	17 (5.1)	

* Copenhagen Burnout Inventory score ≥50 for personal, work-related, and/or client-related burnout.

**Table 2:** Work practices of midwives participating in a survey of midwives in Baden-Württemberg, Germany, 2017 (N=602)

*Work practice*	*All midwives n (%)*	*Midwives with low burnout n (%)*	Midwives with moderate or high burnout* n (%)	*p*
**Total**, n	602	271	331	
**Work model of midwifery**				<0.001
Freelance	273 (45.3)	154 (56.8)	119 (36.0)	
Freelance and hospital affiliation	43 (7.1)	20 (7.4)	23 (6.9)	
Employed	159 (26.4)	55 (20.3)	104 (31.4)	
Freelance and employed	126 (20.9)	41 (15.1)	85 (25.7)	
Freelance, hospital affiliation and employed	1 (0.2)	1 (0.4)	0 (0.0)	
**Work setting**				<0.001
Community based	276 (45.8)	155 (57.2)	121 (36.6)	
Hospital	104 (17.3)	27 (10.0)	77 (23.3)	
Community based and hospital	222 (36.9)	89 (32.8)	133 (40.2)	
**Attending births**				0.12
No	237 (39.4)	116 (42.8)	121 (36.6)	
Yes	365 (60.6)	155 (57.2)	210 (63.4)	
**Weekly work hours**				<0.001
≤24	128 (21.3)	79 (29.2)	49 (14.8)	
25–34	156 (25.9)	68 (25.1)	88 (26.6)	
35–44	169 (28.1)	72 (26.6)	97 (29.3)	
45–54	107 (17.8)	41 (15.1)	66 (19.9)	
≥55	42 (7.0)	11 (4.1)	31 (9.4)	
**Work hours change in past 5 years**				0.003
Substantial increase	21 (3.5)	12 (4.4)	9 (2.7)	
Increase	43 (7.1)	17 (6.3)	26 (7.9)	
Little or no change	90 (15.0)	52 (19.2)	38 (11.5)	
Decrease	174 (28.9)	89 (32.8)	85 (25.7)	
Substantial decrease	207 (34.4)	77 (28.4)	130 (39.3)	
Did not work as midwife 5 years ago	67 (11.1)	24 (8.9)	43 (13.0)	
**Workload change past 5 years**				<0.001
Substantial increase	5 (0.8)	3 (1.1)	2 (0.6)	
Increase	22 (3.7)	14 (5.2)	8 (2.4)	
Little or no change	66 (11.0)	47 (17.3)	19 (5.7)	
Decrease	176 (29.2)	105 (38.7)	71 (21.5)	
Substantial decrease	260 (43.2)	75 (27.7)	185 (55.9)	
Did not work as midwife 5 years ago	73 (12.1)	27 (10.0)	46 (13.9)	
**Focus of practice**				0.58
Rural area	217 (36.0)	103 (38.0)	114 (34.4)	
Small city (<50 K population)	130 (21.6)	57 (21.0)	73 (22.1)	
Medium-sized city (50−100 K population)	113 (18.8)	45 (16.6)	68 (20.5)	
Large city (>100 K population)	142 (23.6)	66 (24.4)	76 (23.0)	

* Copenhagen Burnout Inventory score ≥50 for personal, work-related, and/or client-related burnout.

### Burnout levels and scores

**Figure 1:**
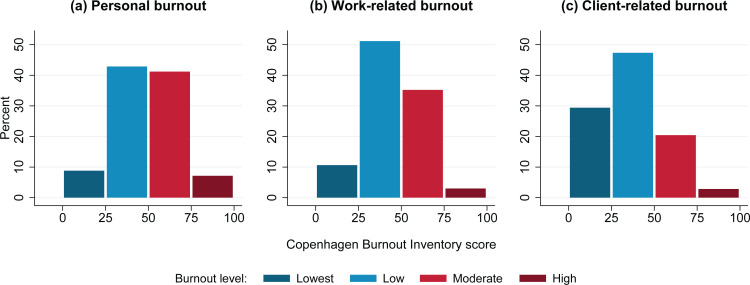
Levels of burnout among midwives in a survey of midwives in Baden-Württemberg, Germany, 2017 (N=602)

In the study sample, 41.2% of midwives had moderate levels of personal burnout and 7.1% had high levels of personal burnout. Related to work, 35.2% of midwives had moderate levels of burnout and 3.0% had high levels of burnout. With respect to clients, 20.4% of midwives had moderate levels of burnout and 2.8% had high levels of burnout. Over half (55.0%) of midwives had moderate or high burnout levels in at least one CBI dimension (Figure 1). Mean CBI dimension scores were 46.4 (SD: 17.6) for personal burnout, 43 (SD: 15.6) for work-related burnout, and 33.8 (SD: 18.7) for client-related burnout. The average score across dimensions was 41.1 (SD: 15.6). Cronbach’s alpha was 0.86 for the personal burnout score, 0.85 for the work-related burnout score, 0.84 for the client-related burnout score, and 0.93 for the overall score. These values appear comparable to Cronbach’s alpha of 0.85–0.87 that was reported when the CBI was introduced^15^. Burnout levels and midwives’ answers to the 19 individual CBI questions are summarized in Supplementary file Tables S1 and S2.

### Correlates of burnout

Midwives with moderate or high burnout in at least one dimension were less likely to be a member of a midwifery association than midwives with low burnout in all dimensions (p=0.008). Overall, the age distribution of midwives with moderate or high burnout in any dimension and midwives with low burnout in all dimensions was similar (p=0.073), but midwives with moderate or high burnout were more likely to be 25–34 years old compared to all other age groups (p=0.004) (Table 1).

When looking at work models, midwives with moderate or high burnout in at least one dimension were more frequently employed or worked both, employed and freelance, than midwives with low burnout levels in all dimensions, who worked more frequently freelance (p<0.001). Compared to midwives with low burnout levels, midwives with moderate or high burnout worked more hours per week (p<0.001), experienced more frequently a substantial decrease of work hours (p=0.003) and workload (p<0.001), or did not work as midwife in the previous 5 years. Midwifes with moderate or high burnout attended births as frequently as midwifes with low burnout levels (p=0.12) (Table 2).

Attitudes toward midwifery correlated with burnout. Midwives with moderate or high burnout were less likely to recommend midwifery as a profession (p<0.001) and more likely to have intentions to leave midwifery (p=0.003) (Table 1).

### Burnout dimensions

**Figure 2:**
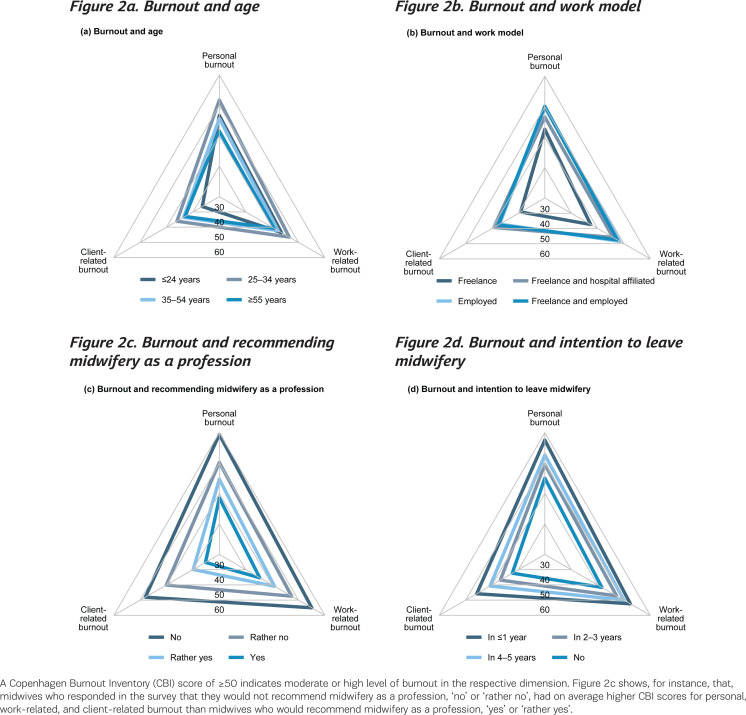
Burnout dimensions of the Copenhagen Burnout Inventory and their relationship to midwives’ age, work model, and attitudes toward midwifery in a survey of midwives in Baden-Württemberg, Germany, 2017 (N=602)

The mean scores for personal burnout (p<0.001) and work-related burnout (p=0.026) differed across age groups; the mean score for client-related burnout did not (p=015). The mean personal and work-related burnout scores were highest for midwives aged 25–34 years. Mean scores in all three burnout dimensions assessed by the CBI differed with midwives' work model, their recommendation of midwifery as a profession, and their intention to leave midwifery (all p<0.001). Midwives working freelance only had lower mean scores for personal, work-related, and client-related burnout than midwives working employed or freelance and employed (all p<0.001). Midwives not or rather not recommending the midwifery as a profession had higher mean scores in all burnout dimensions compared to those recommending the midwifery profession (all p<0.001). Midwives not intending to leave the profession had higher mean scores in all burnout dimensions compared to those with any intention to leave midwifery in the next 5 years (all p≤0.004) (Figure 2).

### Relationship of attitudes toward midwifery with burnout and midwives’ characteristics and work practices

In univariable regressions, having moderate or high levels in personal burnout, work-related burnout, and/or client-related burnout was significantly associated with a lower likelihood to recommend midwifery as a profession (OR=0.33; 95% CI: 0.23–0.46) and an increased likelihood to have the intention to leave midwifery (OR=2.53; 95% CI: 1.59–4.0). Working ≥55 hours per week was significantly associated with a lower likelihood to recommend midwifery as a profession (OR=0.24; 95% CI: 0.11–0.49). Attending births (OR=0.51; 95% CI: 0.34–0.78) and having an age of ≥55 years (OR=5.55; 95% CI: 1.5–20) were both significantly associated with the intention to leave midwifery (Table 3, columns 1 and 3).

**Table 3:** Relationship of recommending midwifery as a profession and intention to leave midwifery with burnout, midwife characteristics, and work practices in a survey of midwives in Baden-Württemberg, Germany, 2017 (N=602)

*Covariate*	*Recommending midwifery (yes/no)*		*Intention to leave midwifery (yes/no)*	
	*Univariable regression OR (95% CI)*	*Multivariable regression OR (95% CI)^#^*	*Univariable regression OR (95% CI)*	*Multivariable regression OR (95% CI)^#^*
**Burnout level**				
Low^†^ (Ref.)	1	1	1	1
Moderate or high^‡^	0.33 (0.23–0.46)***	0.34 (0.23–0.49)***	2.53 (1.59–4.0)***	3.39 (2.0–5.9)***
**Age** (years)				
≤24 (Ref.)	1	1	1	1
25–34	0.71 (0.27–1.9)	0.75 (0.26–2.2)	0.78 (0.21–3.0)	0.58 (0.14–2.4)
35–44	0.64 (0.24–1.7)	0.56 (0.19–1.6)	0.66 (0.18–2.5)	0.53 (0.12–2.2)
45–55	0.94 (0.36–2.5)	0.88 (0.31–2.5)	0.80 (0.22–2.9)	0.69 (0.17–2.8)
≥55	0.65 (0.24–1.8)	0.55 (0.18–1.7)	5.55 (1.5–20)**	4.97 (1.2–21)*
**Education level** (years)				
9–10 (Ref.)	1	1	1	1
12–13	0.87 (0.6–1.3)	0.83 (0.55–1.3)	0.76 (0.48–1.2)	1.02 (0.59–1.8)
Midwifery studies	0.87 (0.36–2.1)	0.97 (0.37–2.5)	0.39 (0.09–1.7)	0.56 (0.12–2.7)
Other studies	1.79 (0.78–4.1)	1.62 (0.67–3.9)	2.03 (0.9–4.6)	1.90 (0.71–5.1)
**Midwifery association member**				
No (Ref.)	1	1	1	1
Yes	0.95 (0.48–1.9)	0.70 (0.32–1.5)	0.91 (0.39–2.1)	0.84 (0.3–2.3)
**Focus of practice**				
Rural area (Ref.)	1	1	1	1
Small city (<50 K population)	0.90 (0.58–1.4)	0.84 (0.52–1.4)	0.88 (0.49–1.6)	0.90 (0.46–1.8)
Medium-sized city (50−100 K population)	0.92 (0.58–1.5)	0.96 (0.57–1.6)	0.92 (0.5–1.7)	0.77 (0.37–1.6)
Large city (>100 K population)	0.82 (0.54–1.3)	0.69 (0.42–1.1)	1.36 (0.8–2.3)	1.40 (0.74–2.7)
**Work model**				
Freelance (Ref.)	1	1	1	1
Freelance and hospital affiliated	0.54 (0.28–1)	0.76 (0.28– 2.1)	0.38 (0.11–1.3)	0.73 (0.15–3.6)
Employed	0.80 (0.54–1.2)	1.12 (0.48–2.6)	1.08 (0.64–1.8)	1.47 (0.45–4.8)
Freelance and employed	0.81 (0.53–1.2)	1.24 (0.55–2.8)	1.57 (0.93–2.6)	1.93 (0.62–6.1)
**Work setting**				
Community based (Ref.)	1	1	1	1
Hospital	0.71 (0.45–1.1)	0.81 (0.31–2.1)	0.93 (0.51–1.7)	0.80 (0.21–3.0)
Community based and hospital	0.75 (0.52–1.1)	0.85 (0.38–1.9)	1.08 (0.68–1.7)	1.03 (0.33–3.2)
**Attending births**				
No (Ref.)	1	1	1	1
Yes	0.90 (0.64–1.3)	1.06 (0.69–1.6)	0.51 (0.34–0.78)**	0.53 (0.3–0.93)*
**Weekly work hours** (hours)				
≤24 (Ref.)	1	1	1	1
25–34	0.68 (0.42–1.1)	0.79 (0.47–1.4)	0.75 (0.41–1.4)	0.51 (0.25–1.1)
35–44	0.74 (0.45–1.2)	0.88 (0.52–1.5)	0.78 (0.43–1.4)	0.60 (0.3–1.2)
45–54	0.50 (0.29–0.85)*	0.60 (0.33–1.1)	0.79 (0.41–1.5)	0.53 (0.24–1.2)
≥55	0.24 (0.11–0.49)***	0.31 (0.14–0.7)**	1.07 (0.46–2.5)	0.92 (0.33–2.5)
Constant	Yes	8.73 (2.1–37)**	Yes	0.19 (0.03–1.2)

# All covariates included in the regression model are listed in the table.

† Copenhagen Burnout Inventory (CBI) score <50 for personal, work-related, and client-related burnout.

‡ CBI score ≥50 for personal, work-related, and/or client-related burnout.

* p<0.1,

** p<0.05,

*** p<0.01.

Regression analyses of the relationship of midwives' CBI score and burnout level with midwife characteristics and work practices are provided in Supplementary file Table S3.

After adjusting for midwives’ characteristics and work practices in multivariable regression, moderate or high burnout levels in at least one dimension remained significantly associated with a lower likelihood to recommend midwifery as a profession (OR=0.34; 95% CI: 0.23–0.49) and an increased likelihood to have the intention to leave midwifery (OR=3.39; 95% CI: 2.0–5.9). Working ≥55 hours per week remained significantly associated with a lower likelihood to recommend midwifery as a profession (OR=0.31; 95% CI: 0.14–0.7). Attending births and having and an age of ≥55 years ceased to be significantly associated with the intention to leave midwifery after adjusting for other midwife characteristics and work practices (Table 3, columns 2 and 4).

## DISCUSSION

Based on data from a cross-sectional online survey, we studied levels of burnout among German midwives, correlates of burnout, and the relationship of burnout, midwife characteristics, and work practices with attitudes toward midwifery. Personal burnout, work-related burnout, and client-related burnout were assessed using the CBI. Burnout symptoms were common among study participants. Moderate to high levels of personal burnout and work-related burnout were present in 48.3% and 38.2% of midwives, respectively. Client-related burnout was less common, with moderate to high levels in 23.3% of midwives. Midwives with low burnout levels worked more commonly as freelance midwives and community based. Midwives with moderate or high burnout levels worked more weekly hours and were less likely to be a member of a midwifery association. Moderate or high burnout levels in at least one dimension significantly decreased the likelihood to recommend midwifery as a profession and increased the likelihood to have the intention to leave midwifery. As midwives with burnout appear more likely to leave the profession and more likely to discourage others from choosing the midwifery profession, burnout could be or become a challenge to the midwifery profession in addition to being a risk factor for midwives’ personal health.

While we conducted, to our knowledge, the first assessment of burnout dimension levels among midwives in Germany, previous studies have utilized the CBI to assess the levels of burnout among midwives in Australia^3,16-19,33,34^, New Zealand^20^, Norway^13^, Sweden^21^, Denmark^15,22^, Jordan^35^, the United Kingdom^6^, Ireland^36^, Lithuania^29^, and Canada^12^. These studies found levels of personal burnout and work-related burnout that were comparable to our results. Mean CBI scores for personal burnout ranged from 37.6 in a cross-sectional random sample of 50 midwives in Denmark^22^ to 68.27 in a cross-sectional convenience sample of 321 Jordanian midwives^35^. Work-related burnout levels ranged from 33.85 in a convenience sample of 475 Swedish midwives^21^ to 67.55 in the study among 321 Jordanian midwives^35^. Just like in our study, client-related burnout levels were lower than personal burnout and work-related burnout levels, with a mean score ranging from 8.3 in a convenience sample of 214 Australian midwives working in caseload care models to 60.93 among Jordanian midwives^35^. A meta-analysis on the prevalence of burnout among midwives concluded that moderate or high personal burnout was present in 50% of midwives, moderate or high work-related burnout in 40% of midwives, and moderate or high client-related burnout in 10% of midwives^9^, which compare to 48.3%, 38.2%, and 23.3%, respectively, in our study. The client-related burnout rate of 23.3% in our study was higher than the average estimated in the meta-analysis^9^.

We found that midwives with moderate or high burnout levels were more commonly employed or worked both, employed and freelance, and worked more commonly in the hospital or both, in the hospital and community based. In turn, midwives with low burnout levels worked more commonly freelance and community based. Previous studies have outlined that working models, which are associated with employed and hospital-based work, that is, shift-based work that is fragmented in antenatal care, labor, and postnatal care, come with higher degrees of burnout^16,17,20,22,34^. In contrast, working models that are comparable to freelance and community-based models (e.g. caseload midwifery with continuity of care) not only have a positive impact on client outcomes such as maternal mortality^23^, but are also associated with lower levels of burnout and better mental health among midwives^16,17,20,22,34^. For example, a study on mental wellbeing and burnout among 1073 New Zealand midwives found significantly higher personal burnout scores and work-related burnout scores in employed midwives compared to freelance midwives^20^. The difference in burnout levels was explained by fewer resources, less autonomy, less empowerment, and less professional recognition among employed compared to freelance midwives^20^. Another study from the UK found that hospital-based midwifery practice was associated with less occupational autonomy, lower levels of job satisfaction, and more frequent bullying and/or harassment compared to community-based work^37^, all of which may contribute to higher burnout levels.

In our study, we found an association between burnout and younger age only for the second youngest age group of 25–34 year-old midwives. Previous systematic reviews of studies that either used the CBI^9^ or the Maslach Burnout Inventory^8^ indicate that higher burnout levels in younger midwives are common without further disaggregating younger ages. Remarkably, we did not find differences in burnout levels between midwives who attended births and those who did not. This is particularly interesting against the background that 75% of freelance midwives chose not to attend births in Germany in 2020^32^. Our findings thus indicate that reasons other than higher burnout levels might contribute to the decision not to attend births.

We found that midwives with moderate or high burnout levels in at least one dimension were less likely to recommend midwifery as a profession, and more likely to have an intention to leave midwifery. This confirms findings from a survey among 158 Canadian midwives, where burnout levels were significantly higher among the 34.7% of participants who seriously considered leaving the profession in the previous 12 months^12^. Notably, the question’s wording differed between the Canadian study and our study, and we did not assess reasons for midwives’ intentions to leave their profession. Canadian midwives cited the negative impact of on-call shifts on personal life, their mental health, and their physical health, as reasons for the serious consideration to leave midwifery^12^. In a study among 1037 Australian midwives^38^ and a study among 726 Dutch midwives^39^, 42.8% and 33.7% of participants, respectively, stated that they considered leaving midwifery in the previous 6 months, compared with 17.6% of midwives who had an intention to leave midwifery within the next 5 years in our study. In both former studies, the most common reason for considering leaving midwifery was the dissatisfaction with organization of midwifery care^38,39^. In another study with a sample of 475 Swedish midwives, 30.3% had experienced a situation that made them consider leaving their work. The most common reason was the lack of staff and resources and a stressful work environment, which was also associated with higher CBI dimension scores^21^.

### Limitations

This study is subject to limitations. First, the survey was promoted through the network of the Midwifery Association Baden-Württemberg. Participating midwives may have considered that this study can be used to underscore a need to improve working conditions in midwifery, which could have biased the given answers. Second, most midwives in our sample worked in the state of Baden-Württemberg. This might limit the validity of our findings to other German states, even though midwifery care is organized similarly across federal states in Germany^30^. Third, we collected data from a convenience sample of midwives, which may have introduced a selection bias to our study. On the one hand, midwives that are interested in the topic and/or are affected by their working conditions might have been more likely to participate in the survey. On the other hand, midwives who suffer from high levels of burnout might have already left midwifery or were less likely to participate in the study, even though the survey was in principle open to active and non-active midwives. Fourth, the survey was conducted in 2017 before the COVID-19 pandemic, which changed practices in midwifery and obstetrics^40^. Finally, like previous studies on burnout among midwives^17,20-22,34,37^, we used a cross-sectional study design, which does not allow for the inference of causal relationships.

## CONCLUSIONS

This cross-sectional study assessed the prevalence and level of burnout among midwives in Germany. Using the CBI, we found that 48.3% of midwives reported moderate or high levels of personal burnout and 38.2% of midwives reported moderate or high levels of work-related burnout. Client-related burnout was less common, with 23.3% of midwives reporting moderate or high levels. Midwives with moderate or high burnout were more commonly employed and worked in the hospital compared to midwives with low burnout. Burnout not only appeared to be a common health risk among midwives, but midwives with moderate or high burnout levels were less likely to recommend midwifery as a profession and were more likely to have intentions to leave midwifery. Work practices that reduce burnout among midwives could thus improve the health of individual midwives and strengthen the midwifery workforce.

## Data Availability

The data supporting this research are available from the heiDATA data repository at https://doi.org/10.11588/data/ZEWLBS.
